# Elevated pulmonary vascular resistance is associated with increased lung transplant waitlist mortality among patients with chronic obstructive pulmonary disease and pulmonary hypertension: a retrospective cohort analysis

**DOI:** 10.1186/s12931-024-02674-9

**Published:** 2024-02-07

**Authors:** Shameek K. Gayen, Mary Zulty, Gerard J. Criner

**Affiliations:** Department of Thoracic Medicine and Surgery, Lewis Katz School of Medicine at Temple University Hospital, 3401 N Broad Street, Philadelphia, PA 19140 USA

**Keywords:** COPD, Pulmonary hypertension, Lung transplant, Pulmonary vascular resistance

## Abstract

**Background:**

The latest European Society of Cardiology and European Respiratory Society guidelines have changed the definition of both pre-capillary pulmonary hypertension (PH) and severe PH in chronic lung disease. The clinical significance of these new criteria are unclear among patients with chronic obstructive pulmonary disease (COPD)-PH. We aim to examine the clinical significance of the new PH definitions with regards to lung transplant waitlist mortality amongst patients with COPD-PH.

**Methods:**

This was a retrospective cohort study of adult patients with COPD-PH listed for lung transplantation. Kaplan–Meier survival analyses were performed comparing patients with newly defined pre-capillary PH to those without pre-capillary PH and comparing patients with severe PH, defined as pulmonary vascular resistance (PVR) > 5 WU, to those without severe PH. Both mean pulmonary artery pressure (mPAP) and PVR were analyzed for potential cut-off points associated with increased waitlist mortality. Predictors of waitlist mortality were identified via Cox regression.

**Results:**

Among 6495 patients with COPD-PH listed for lung transplantation, pre-capillary PH was not associated with increased waitlist mortality (logrank p = 0.43), while severe PH was (logrank p < 0.001). Both severe PH (HR 1.79, 95% CI 1.22–2.60, p = 0.003) and PVR > 3.9 WU (HR 1.49, 95% CI 1.14–1.95, p = 0.004) were independently and significantly associated with increased waitlist mortality.

**Conclusions:**

PVR may serve as a strong predictor of lung transplant waitlist mortality among patients with COPD-PH as compared to other pulmonary hemodynamic parameters when predicting transplant waitlist mortality.

**Supplementary Information:**

The online version contains supplementary material available at 10.1186/s12931-024-02674-9.

## Introduction

Pulmonary hypertension (PH) is rather common in advanced chronic obstructive pulmonary disease (COPD), with up to 90% of patients with COPD Global Initiative for Chronic Obstructive Lung Disease (GOLD) Stage IV having a mean pulmonary artery pressure (mPAP) > 20 mmHg. [1, 2] In select patient populations of advanced COPD, such as those undergoing evaluation for lung transplantation, PH is highly prevalent, and associated with increased morbidity and mortality. [3]

The presence of PH has a stronger association with mortality in patients with COPD than forced expiratory volume in 1 s (FEV1), while enlarged pulmonary artery diameter on computed tomography (CT) scan is predictive of hospitalization for acute exacerbation of COPD [4, 5]. With regards to patients with COPD undergoing lung transplantation evaluation, both mPAP ≥ 25 mmHg and mPAP ≥ 35 mmHg are significantly associated with increased risk of death. [6]

The latest 2022 European Society of Cardiology (ESC)/European Respiratory Society (ERS) Guidelines for the diagnosis and treatment of pulmonary hypertension redefined the criteria for PH, including PH associated with chronic lung disease [7]. PH is defined as mPAP > 20 mmHg, with pre-capillary PH now defined as mPAP > 20 mmHg, pulmonary artery wedge pressure (PAWP) ≤ 15 mmHg, and pulmonary vascular resistance > 2 Woods units (WU). Post-capillary PH is defined as PAWP > 15 mmHg and PVR ≤ 2 WU, combined pre- and post-capillary PH is defined as PAWP > 15 mmHg and PVR > 2 WU, and unclassified PH is defined as PAWP ≤ 15 mmHg and PVR ≤ 2 WU. Additionally, severe PH is now defined as PVR > 5 WU; it was previously defined as mPAP ≥ 35 mmHg or mPAP ≥ 25 mmHg with cardiac index < 2.5 L/min/m2 [7]. The significance of these new definitions on outcomes in patients with COPD-PH listed for lung transplantation is unclear.

We hypothesize that among patients with COPD-PH listed for lung transplantation, those with pre-capillary PH and those with severe PH as per the latest ESC/ESR guidelines will have decreased waitlist survival or increased risk of lung transplant waitlist mortality. Our primary objective is to determine whether lung transplant waitlist survival is decreased among COPD-PH patients with pre-capillary PH and with severe PH. Our secondary objective is to determine significant associations with transplant waitlist mortality among patients with COPD-PH.

## Methods

### Study design

This was a retrospective review of consecutive lung transplant candidates from the Scientific Registry of Transplant Recipients (SRTR) national database between May 2005 (implementation of Lung Allocation Score, now composite allocation score (CAS)) and December 2022, collected directly by the Organ Procurement and Transplantation Network (OPTN) and overseen by the United Network for Organ Sharing (UNOS) in the United States of America. We restricted our analysis to adult lung transplant candidates (≥ 18 years of age) with the primary diagnosis of COPD and associated PH (mPAP > 20 mm Hg on right heart catheterization) listed for lung transplant; all patients undergoing evaluation for lung transplantation received right heart catheterization. We excluded those listed for heart–lung transplant.

The data reported here have been supplied by the Hennepin Healthcare Research Institute (HHRI) as the contractor for the SRTR. The interpretation and reporting of these data are the responsibility of the author(s) and in no way should be seen as an official policy of or interpretation by the SRTR or the U.S. Government. This study used data from the SRTR. The SRTR data system includes data on all donor, wait-listed candidates, and transplant recipients in the US, submitted by the members of the OPTN. The Health Resources and Services Administration (HRSA), U.S. Department of Health and Human Services provides oversight to the activities of the OPTN and SRTR contractors. The proposed study was approved by the SRTR.

### Data collection

We collected candidate baseline characteristics such as age, sex, blood type, race, body mass index (BMI), and comorbidities. We also collected the available clinical data at time of listing used to calculate the Composite Allocation score (CAS). These include functional vital capacity (FVC), 6-min walk distance (6MWD), pulmonary artery systolic pressure (PASP), mPAP, partial pressure of carbon dioxide (PaCO2), oxygen requirement, creatinine, mechanical ventilation, and functional status at the time of listing; these were also included in the prior Lung Allocation Score (LAS) system [8]. These variables are specifically used to evaluate the expected 1-year waiting list mortality without a transplant and as such were included in our analysis of waitlist mortality. Additionally, FEV1, candidate height, double lung preference, PAWP, cardiac output (CO), and PVR were collected. Patients were categorized as having pre-capillary PH, post-capillary PH, combined pre- and post-capillary PH, or unclassified PH, and as having severe PH (PVR > 5 WU) or not. PaCO2 was collected as a dichotomous variable as defined by CAS cut-off, while FEV1, FVC, and oxygen requirement were collected as dichotomous variables given missing data.

### Statistical analysis

All continuous variables were presented as mean ± standard deviation or median (IQR) unless otherwise stated. The categorical variables were compared using Pearson chi-square test or Fisher’s exact test where applicable. The continuous variables were compared between groups using the Mann–Whitney U test. Kaplan–Meier survival analysis was performed comparing transplant waitlist survival between patients with COPD and pre-capillary PH and patients with COPD and post-capillary, combined pre- and post-capillary, or unclassified PH. Kaplan–Meier survival analysis was also performed comparing transplant waitlist survival between patients with COPD and severe PH and patients with COPD and non-severe PH. Stratification of PH via mPAP and PVR levels via quintiles was performed, with subsequent cox regression and Kaplan–Meier analysis to determine thresholds of waitlist mortality. Univariable with subsequent multivariable cox regression was then performed to identify significant and independent associations with lung transplant waitlist mortality among this cohort. Multicollinearity analysis was performed to assess the degree of correlation between variables utilized in multivariable cox regression and potential effect on regression findings.

## Results

**Fig. 1:**
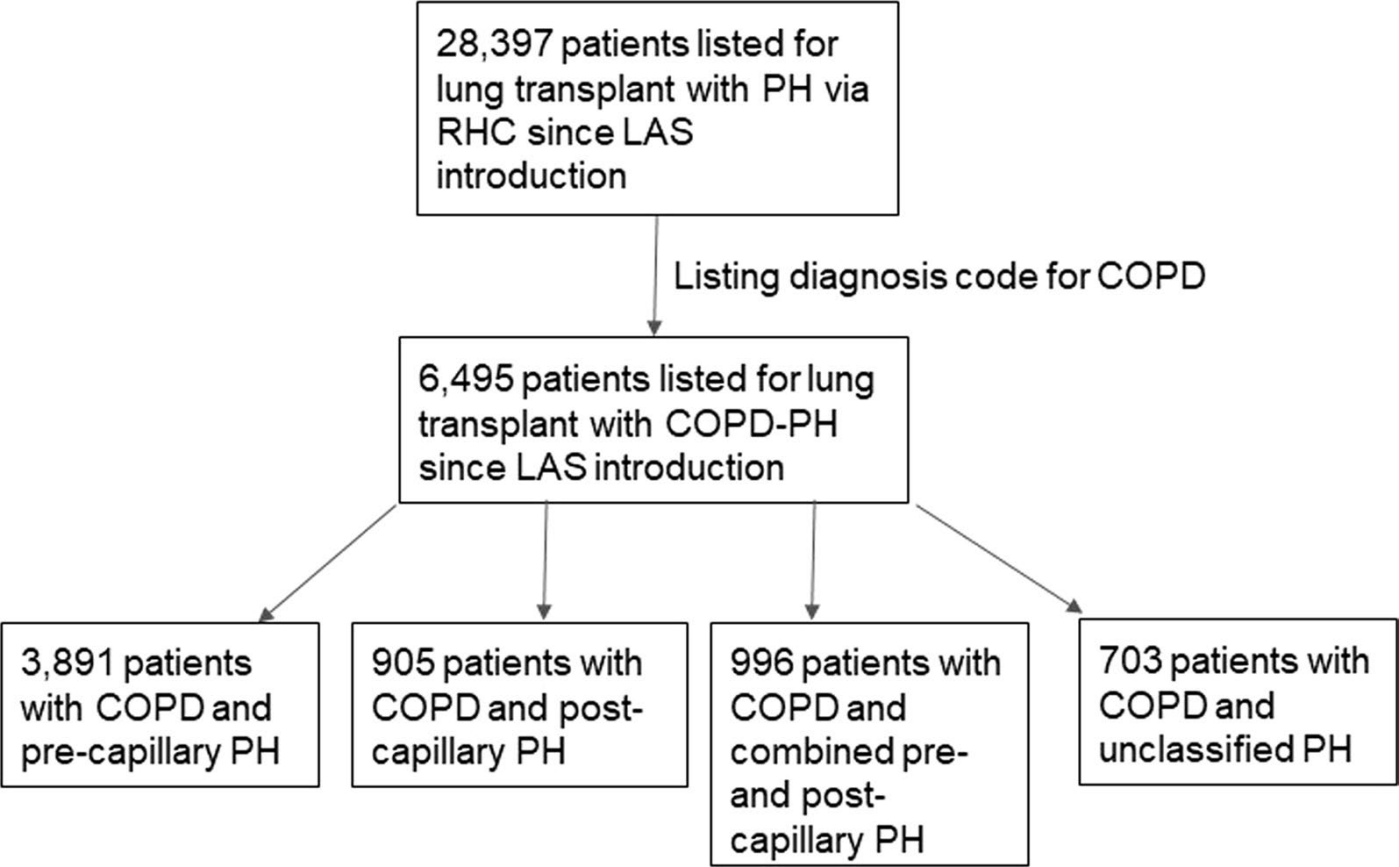
COPD-PH Cohort Patient Selection. Patients with COPD-PH listed for lung transplantation after LAS implementation in May 2005 were selected. PH defined as mPAP > 20 mmHg. Patients were further classified by PH category. Pre-capillary PH is defined as mPAP > 20 mmHg, PAWP ≤ 15 mmHg, and PVR > 2 WU. Post-capillary PH is defined as mPAP > 20 mmHg, PAWP > 15 mmHg, and PVR ≤ 2 WU. Combined pre- and post-capillary PH is defined as mPAP > 20 mmHg, PAWP > 15 mmHg, and PVR > 2 WU. Unclassified PH is defined as mPAP > 20 mmHg, PAWP ≤ 15 mmHg, and PVR ≤ 2 WU. *COPD* Chronic obstructive pulmonary disease**.** *LAS* Lung allocation score. mPAP: Mean pulmonary artery pressure. *PAWP* Pulmonary artery wedge pressure. *PH* Pulmonary hypertension. *PVR* Pulmonary vascular resistance. *RHC* Right heart catheterization. *WU* Woods units

Since May 2005, 6495 patients with COPD-PH have been listed for lung transplantation (Fig. 1). Baseline characteristics and comorbid conditions are seen in Table 1. 1758 patients had an oxygen requirement of at least 3 L/min (median oxygen requirement), while 1345 patients had an oxygen requirement less than 3 L/min. 264 patients did not require any supplemental oxygen, while 3392 patients did not have oxygen requirement recorded. 106 patients had coronary artery disease (CAD), 1008 patients had hypertension, 627 patients had a history of cancer, and 610 patients had diabetes (Table 1).

**Table 1:** Baseline characteristics, n = 6495

Age, years	60.6 ± 6.5
BMI	25.0 ± 4.2
Gender	
Male, n (%)	3116 (48.0)
Female, n (%)	3379 (52.0)
Race	
White, n (%)	5903 (90.9)
Black, n (%)	527 (8.1)
Other, n (%)	65 (1.0)
Coronary Artery Disease	
Yes, n (%)	106 (1.6)
No, n (%)	2814 (43.3)
Unknown, n (%)	3575 (55.1)
Hypertension	
Yes, n (%)	1008 (15.5)
No, n (%)	1980 (30.5)
Unknown, n (%)	3507 (54.0)
History of Cancer, n (%)	627 (9.7)
Diabetes, n (%)	610 (9.4)
Blood Type	
A, n (%)	2698 (41.5)
B, n (%)	705 (10.9)
AB, n (%)	281 (4.3)
O, n (%)	2811 (43.3)
Functional Status	
No assistance needed, n (%)	471 (7.3)
Some assistance needed, n (%)	5811 (89.5)
Total assistance needed, n (%)	170 (2.6)
Unknown, n (%)	43 (0.7)

Baseline characteristics including demographics and comorbid conditions of the entire COPD-PH cohort. *BMI* Body mass index. *COPD* Chronic obstructive pulmonary disease. *PH* Pulmonary hypertension

Among this COPD-PH cohort, 3891 patients (59.9%) had pre-capillary PH, 905 patients (13.9%) had post-capillary PH, 996 patients (15.4%) had combined pre- and post-capillary PH, and 703 patients (10.8%) had unclassified PH (Fig. 1). 543 (8.4%) patients had severe PH as defined by the latest ESC/ESR guidelines (Table 2). In terms of spirometry, 2741 (42.2%) patients had very severe airflow obstruction (FEV1 < 30% predicted) and 1353 patients had FVC < 50% predicted (20.8%). 802 patients (12.3%) had a 6MWD < 150 m. 54 patients (0.8%) required mechanical ventilation while on the lung transplant waitlist, and 3391 patients (52.2%) were listed as double lung preference only (Table 2).

**Table 2:** Clinical characteristics, n = 6495

PASP (mm Hg)	40.6 ± 9.7
mPAP (mm Hg)	28.2 ± 6.3
PAWP (mm Hg)	13.5 ± 4.9
CO (L/min)	5.3 ± 1.4
PVR (WU)	3.0 ± 1.8
Pre-capillary PH, n (%)	3891 (59.9)
Severe PH, n (%)	543 (8.4)
FEV1 (% predicted)	21.7
< 30% predicted, n (%)	2741 (42.2)
≥ 30% predicted, n (%)	378 (5.8)
Unknown	3376 (52.0)
FVC (% predicted)	54.0
< 50% predicted, n (%)	1353 (20.8)
≥ 50% predicted, n (%)	1771 (27.3)
Unknown	3371 (51.9)
6MWD (m)	221.2 ± 113.0
Oxygen requirement (L/min)	3.1 ± 2.5
≥ 3 L/min, n (%)	1758 (27.1)
< 3 L/min, n (%)	1345 (20.7)
Unknown	3392 (52.2)
PaCO2 (mm Hg)	50.2 ± 12.5
≥ 40 mm Hg, n (%)	2278 (35.1)
< 40 mm Hg, n (%)	442 (6.8)
Unknown, n (%)	3775 (58.1)
Mechanical Ventilation, n (%)	54 (0.8)
Double Lung Preference Only, n (%)	3391 (52.2)
Creatinine (mg/dL)	0.82 ± 0.33
≥ 0.8 mg/dL, n (%)	1735 (26.7)
< 0.8 mg/dL, n (%)	1395 (21.5)
Unknown, n (%)	3365 (51.8)

Clinical characteristics including pulmonary hemodynamics via right heart catheterization, pulmonary function test data, and other clinical characteristics that comprise the CAS score. *6MWD* 6-min walk distance. *CAS* Composite lung allocation score. *CO* Cardiac output. *FEV1* Forced expiratory volume in 1 s. *FVC* Functional vital capacity. *mPAP* Mean pulmonary artery pressure. *PaCO2* Partial pressure of carbon dioxide. *PASP* Pulmonary artery systolic pressure. *PAWP* Pulmonary artery wedge pressure. *PVR* Pulmonary vascular resistance

Among this COPD-PH cohort, 668 patients (10.3%) died on the waitlist, while 5827 patients (89.7%) underwent lung transplantation (Table 3). Patients who underwent lung transplantation spent 7.4 months on the waitlist and patients who died on the waitlist spent 17.5 months on the waitlist (p < 0.001). A similar proportion of patients with COPD and pre-capillary PH died prior to lung transplantation as compared to those with COPD and post-capillary or combined pre- and post-capillary PH. Among patients with COPD anPH (or PVR > 5 WU), 16.6% died prior to lung transplantation as compared to 9.7% of patients with COPD and non-severe PH (p < 0.001).. Patients with COPD and severe PH spent less time on the transplant waitlist, but a higher proportion died prior to lung transplant as compared to patients with COPD and non-severe PH (Table 4).

**Table 3:** Outcomes

	Transplant waitlist death (n = 668)	Lung transplantation (n = 5827)
Time on Waitlist (months)*	17.5 ± 8.6	7.4 ± 5.5
Pre-capillary PH (n = 3891) vs rest (n = 2604)		
Pre-Capillary PH, n (%)	406 out of 3891 (10.4)	3485 out of 3891 (89.6)
Post- or combined pre- and post-capillary PH, n (%)	262 out 2604 (10.1)	2342 out of 2604 (89.9)
Severe PH (n = 543) vs non-severe PH (n = 5952)*		
Severe PH, n (%)	90 out of 543 (16.6)	453 out of 543 (83.4)
Non-severe PH, n (%)	578 out of 5952 (9.7)	5374 out of 5952 (90.3)

Patients with COPD-PH who died on the lung transplant waitlist spent significantly longer time on the waitlist than those who received lung transplantation. A significantly higher proportion of patients with severe PH, as defined by PVR > 5 WU, died on the transplant waitlist as compared to those without severe PH. *COPD* Chronic obstructive pulmonary disease. *PH* Pulmonary hypertension

*p < 0.05

**Table 4:** Outcomes stratified by pulmonary hypertension severity

	Severe PH, n (%) (n = 543)	Non-severe PH, n (%) (n = 5952)
Mean Time on Waitlist (months)*	5.9 ± 8.6	8.6 ± 13.0
Median Time on Waitlist, months (IQR)*	2.47 (0.69–8.02)	3.52 (1.08–10.45)
Pre-capillary PH (n = 3891) vs rest (n = 2604)		
Pre-Capillary PH, n (%)	472 out of 3891 (12.1)	3419 out of 3891 (87.9)
Post- or combined pre- and post-capillary PH, n (%)	71 out of 2604 (2.7)	2533 out of 2604 (97.3)
Transplant Waitlist Death (n = 668)*	90 out of 543 (16.6)	578 out of 5952 (9.7)
Lung Transplantation (n = 5827)*	453 out of 543 (83.4)	5374 out of 5952 (90.3)

Patients with COPD and severe PH (defined as PVR > 5 WU) had longer time on the waitlist along with higher mortality rate than those with COPD and non-severe PH. *COPD* Chronic obstructive pulmonary disease. *IQR* Interquartile range. *PH* Pulmonary hypertension. *PVR* Pulmonary vascular resistance

*p < 0.05

**Fig. 2:**
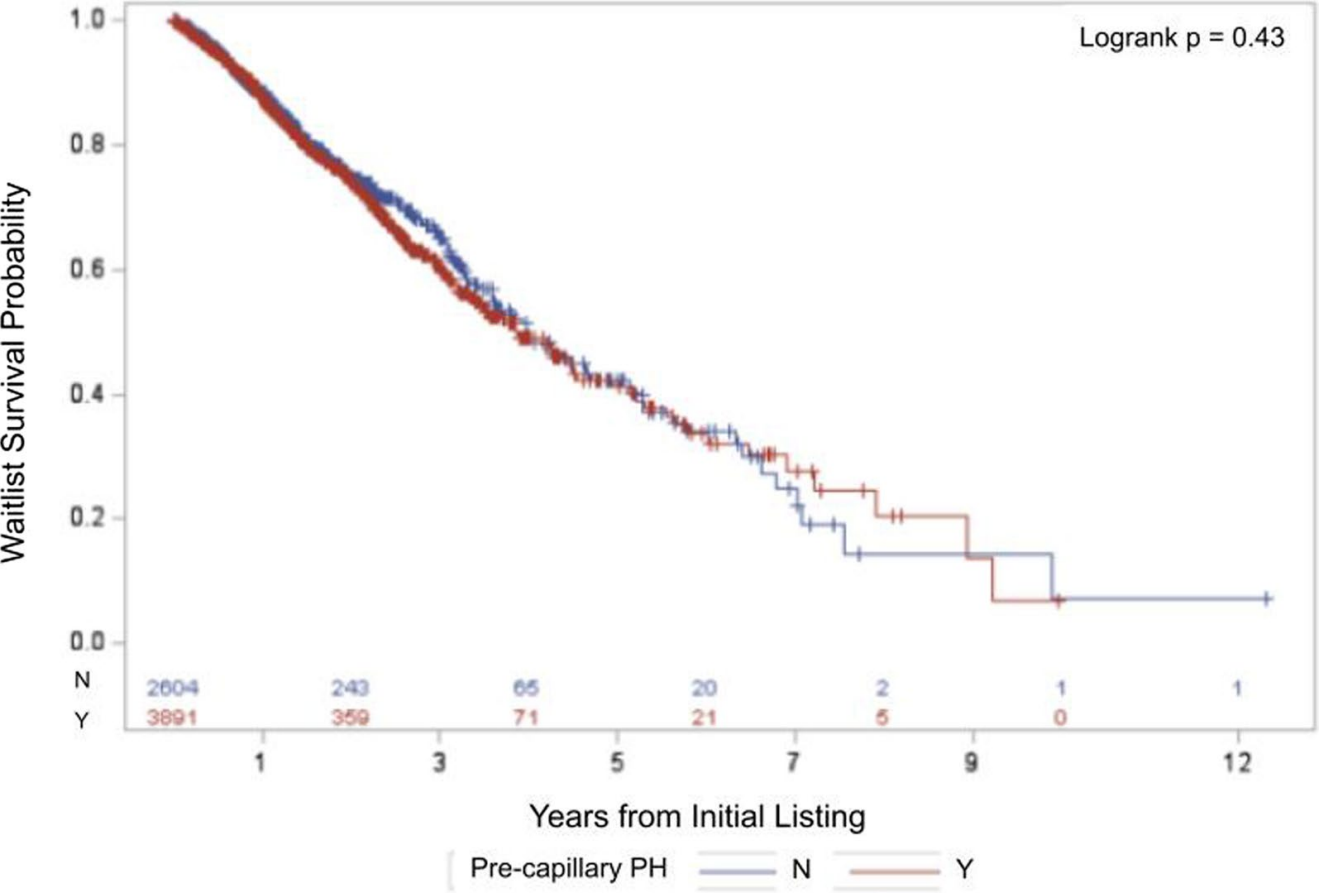
Kaplan–Meier Survival Analysis Comparing Pre-Capillary Pulmonary Hypertension to Rest. Kaplan Meier survival analysis comparing COPD-PH patients with pre-capillary PH (defined as mPAP > 20 mmHg, PAWP ≤ 15 mmHg, and PVR ≥ 2 WU), to those without pre-capillary PH, encompassing post-capillary PH, combined pre- and post-capillary PH, and unclassified PH. Post-capillary PH is defined as mPAP > 20 mmHg, PAWP > 15 mmHg, and PVR ≤ 2 WU. Combined pre- and post-capillary PH is defined as mPAP > 20 mmHg, PAWP > 15 mmHg, and PVR > 2 WU. Unclassified PH is defined as mPAP > 20 mmHg, PAWP ≤ 15 mmHg, and PVR ≤ 2 WU. Similar transplant waitlist survival probability observed between the two groups. Number of COPD-PH patients in each group at risk of death displayed on the bottom, with changes in curves reflective of patients who suffered waitlist mortality. *COPD* Chronic obstructive pulmonary disease. *mPAP* Mean pulmonary artery pressure. *PAWP* Pulmonary artery wedge pressure. *PH* Pulmonary hypertension. *PVR* Pulmonary vascular resistance

**Fig. 3:**
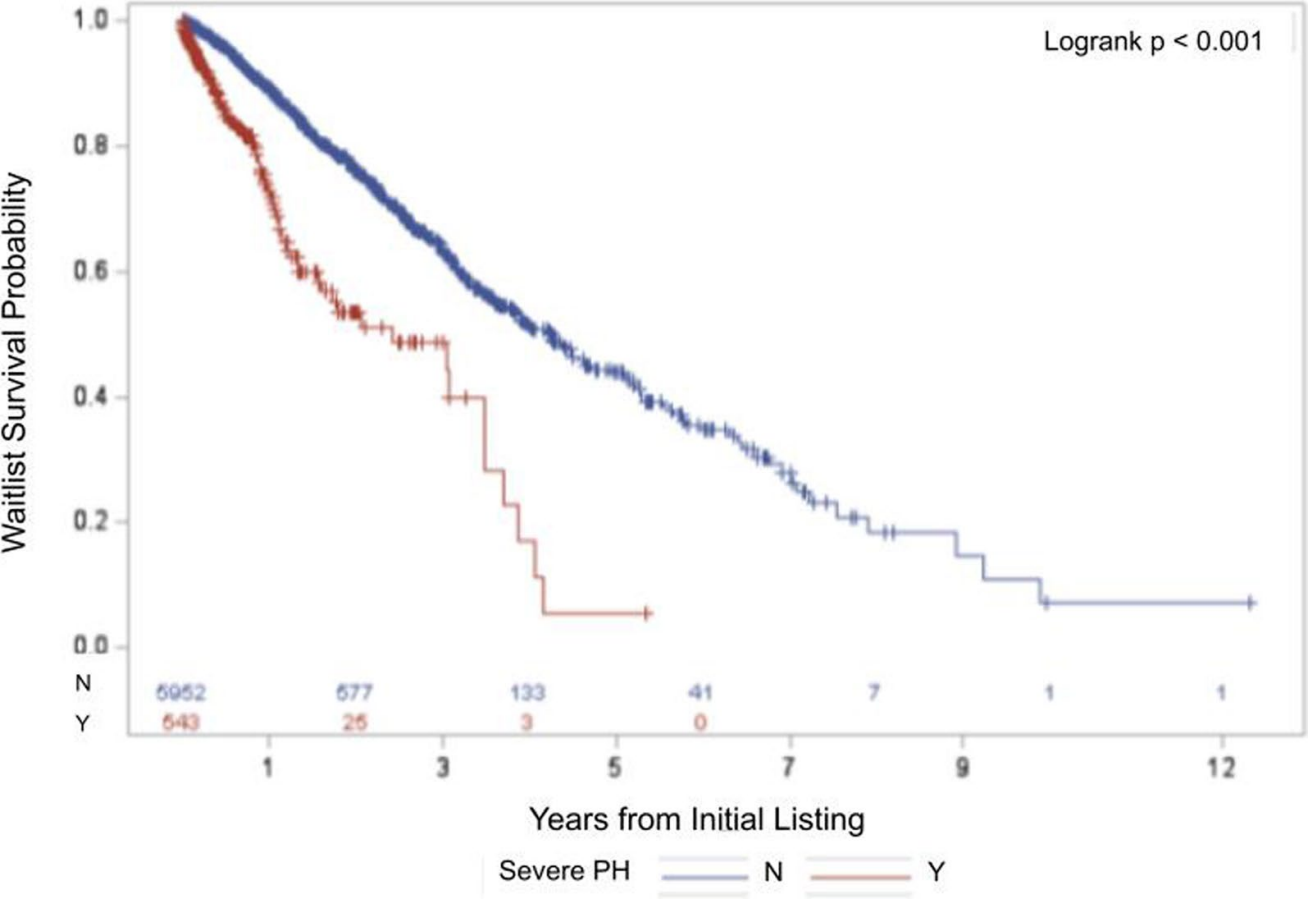
Kaplan–Meier Survival Analysis Comparing Severe PH to Non-Severe PH. Kaplan Meier survival analysis comparing COPD-PH patients with severe PH (defined as PVR > 5 WU), to those without severe PH. Significantly decreased transplant waitlist survival probability observed among those with severe PH. Number of COPD-PH patients in each group at risk of death displayed on the bottom, with changes in curves reflective of patients who suffered waitlist mortality. *COPD* Chronic obstructive pulmonary disease. *PH* Pulmonary hypertension. *PVR* Pulmonary vascular resistance

Kaplan–Meier analysis showed similar transplant waitlist survival probability among COPD patients with pre-capillary PH and COPD patients with post-capillary or combined pre- and post-capillary PH (logrank p = 0.43; Fig. 2). Kaplan–Meier analysis showed significantly decreased waitlist survival among patients with COPD and severe PH as compared to those with COPD and non-severe PH (logrank p < 0.001; Fig. 3).

Quintiles for both mPAP and PVR distributions in this cohort were determined (Table 5). For mPAP, the quintiles were determined to be less than 23 mmHg, 23 to < 25 mmHg, 25 to < 28 mmHg, 28 to < 32 mmHg, and ≥ 32 mmHg. For PVR, the quintiles were determined to be less than 1.86 WU, 1.86 to < 2.42 WU, 2.42 to < 3.01 WU, 3.01 to < 3.90 WU, and ≥ 3.9 WU. Among mPAP quintiles, mPAP ≥ 32 mmHg (HR 1.96, 95% CI 1.53–2.50, p < 0.001) was associated with increased risk of waitlist mortality (Table 5). Among PVR quintiles, PVR ≥ 3.9 WU (HR 1.86, 95% CI 1.46–2.36, p < 0.001) was associated with increased risk of waitlist mortality (Table 5).

**Table 5:** Quintile distribution of mean pulmonary artery pressure and pulmonary vascular resistance and risk of waitlist mortality

	Number of patients	Risk of waitlist mortality
mPAP	Total n = 6495	
< 23 mmHg	988	Reference
23 to < 25 mmHg	1089	HR 0.87, 95% CI 0.65–1.15, p = 0.33
25 to < 28 mmHg	1569	HR 1.09, 95% CI 0.84–1.41, p = 0.53
28 to < 32 mmHg	1410	HR 1.19, 95% CI 0.92–1.54, p = 0.18
≥ 32 mmHg	1439	HR 1.96, 95% CI 1.53–2.50, p < 0.001*
PVR	Total n = 6495	
< 1.86 WU	1299	Reference
1.86 to < 2.42 WU	1291	HR 1.13, 95% CI 0.87–1.45, p = 0.36
2.42 to < 3.01 WU	1305	HR 1.01, 95% CI 0.79–1.31, p = 0.92
3.01 to < 3.90 WU	1301	HR 1.21, 95% CI 0.94–1.55, p = 0.14
≥ 3.90 WU	1299	HR 1.86, 95% CI 1.46–2.36, p < 0.001*

Hazard ratios express risk of waitlist mortality in relation to the reference group. Increased risk of mortality seen in patients with mPAP ≥ 32 mmHg and in patients with PVR ≥ 3.9 WU. *HR* Hazard ratio. *mPAP* mean pulmonary artery pressure. *PVR* pulmonary vascular resistance. *WU* Woods units

*Statistically significant association with increased risk of lung transplant waitlist mortality

Univariable cox regression determined that age, body mass index (BMI), CAD, hypertension, diabetes, double lung preference, blood type A, FVC, both new and old definitions of severe PH, mPAP ≥ 32 mmHg (identified from quintile analysis), PVR ≥ 3.9 WU (identified from quintile analysis), mechanical ventilation, oxygen requirement, PASP, and 6MWD were significantly associated with the risk of waitlist mortality (Table 6). These variables were subsequently utilized in multivariable cox regression to determine significant and independent associations with waitlist mortality. Age, CAD, hypertension, PASP, mechanical ventilation, double lung preference, new criteria for severe PH (or PVR > 5 WU), and PVR ≥ 3.9 WU were significantly and independently associated with increased risk of transplant waitlist mortality among patients with COPD-PH listed for lung transplantation (Table 6). BMI, 6MWD, and blood type A were significantly and independently associated with reduced risk of transplant waitlist mortality (Table 6). Of the pulmonary hemodynamic variables, only PASP and the PVR cut-offs maintained significance after multivariable cox regression; both PVR > 5 WU (HR 1.79, 95% CI 1.22–2.60, p = 0.003) and PVR ≥ 3.9 WU (HR 1.49, 95% CI 1.14–1.95, p = 0.004) have a stronger association with waitlist mortality than PASP (HR 1.02, 95% CI 1.01–1.03, p < 0.0001) and the older, mPAP-based classification of severe PH (HR 0.89, 95% CI 0.67–1.18, p = 0.42).

**Table 6:** Predictors of waitlist mortality analysis

	Univariable	Multivariable
Age	HR 1.05, 95% CI 1.03–1.06, p < 0.001*	HR 1.04, 95% CI 1.02–1.06, p < 0.001**
Gender	HR 1.13, 95% CI 0.97–1.32, p = 0.11	
BMI	HR 0.97, 95% CI 0.95–0.99, p = 0.001*	HR 0.96, 95% CI 0.94–0.99, p = 0.002**
Race	Black: HR 1.26, 95% CI 0.96–1.65, p = 0.10 Other: HR 0.35, 95% CI 0.11–1.08, p = 0.07	
CAD	Yes: HR 2.08, 95% CI 1.31–3.30, p = 0.002* Unknown: HR 1.02, 95% CI 0.87–1.20, p = 0.80	Yes: HR 1.78, 95% CI 1.07–2.94, p = 0.03** Unknown: HR 0.38, 95% CI 0.09–1.62, p = 0.19
Hypertension	Yes: HR 1.42, 95% CI 1.16–1.73, p = 0.001* Unknown: HR 1.19, 95% CI 0.99–1.42, p = 0.06	Yes: HR 1.45, 95% CI 1.17–1.80, p = 0.001** Unknown: HR 1.46, 95% CI 1.05–2.02, p = 0.02**
Cancer	HR 1.15, 95% CI 0.88–1.49, p = 0.32	
Diabetes	HR 1.56, 95% CI 1.23–1.98, p < 0.001*	HR 1.35, 95% CI 0.98–1.86, p = 0.06
Creatinine	> 0.8: HR 1.06, 95% CI 0.88–1.29, p = 0.53 Unknown: HR 1.05, 95% CI 0.87–1.27, p = 0.62	
Candidate Height	HR 0.99, 95% CI 0.99–1.01, p = 0.62	
Blood Type	A: HR 0.80, 95% CI 0.68–0.95, p = 0.01* AB: HR 1.37, 95% CI 0.85–2.21, p = 0.19 B: HR 0.87, 95% CI 0.65–1.17, p = 0.37	A: HR 0.79, 95% CI 0.64–0.98, p = 0.04** AB: HR 1.32, 95% CI 0.75–2.33, p = 0.34 B: HR 0.72, 95% CI 0.48–1.07, p = 0.11
Functional Status	Some assistance: HR 1.15, 95% CI 0.86–1.53, p = 0.35 Total assistance: HR 1.41, 95% CI 0.84–2.37, p = 0.20 Unknown: HR 1.18, 95% CI 0.47–2.95, p = 0.73	
O2 requirement	> 3L: HR 1.27, 95% CI 1.04–1.55, p = 0.02* Unknown: HR 1.25, 95% CI 1.03–1.51, p = 0.02	> 3L: HR 1.17, 95% CI 0.95–1.43, p = 0.14 Unknown: HR 1.45, 95% CI 0.78–2.71, p = 0.24
PaCO2	> 40 mm Hg: HR 0.97, 95% CI 0.74–1.28, p = 0.84 Unknown: HR 1.08, 95% CI 0.82–1.42, p = 0.58	
PASP	HR 1.03, 95% CI 1.02–1.04, p < 0.001*	HR 1.02, 95% CI 1.01–1.03, p < 0.001**
PAWP	HR 1.01, 95% CI 0.99–1.02, p = 0.33	
CO	HR 0.99, 95% CI 0.92–1.03, p = 0.27	
Pre-capillary PH	HR 1.07, 95% CI 0.91–1.24, p = 0.43	
Severe PH new (PVR > 5 WU)	HR 2.73, 95% CI 2.19–3.42, p < 0.001*	HR 1.79, 95% CI 1.22–2.60, p = 0.003**
Severe PH old (mPAP > 35 mmHg or mPAP > 25 mmHg with CI < 2.5 L/min/m2)	HR 1.58, 95% CI 1.33–1.87, p < 0.001*	HR 0.89, 95% CI 0.67–1.18, p = 0.42
mPAP ≥ 32 mmHg	HR 1.87, 95% CI 1.58–2.22, p < 0.001*	HR 1.13, 95% CI 0.82–1.56, p = 0.45
PVR ≥ 3.9 WU	HR 1.71, 95% CI 1.44–2.04, p < 0.001*	HR 1.49, 95% CI 1.14–1.95, p = 0.004**
6MWD	HR 0.98, 95% CI 0.97–0.99, p < 0.001*	HR 0.97, 95% CI 0.96–0.98, p < 0.001**
FEV1	FEV1 < 30%: HR 0.99, 95% CI 0.75–1.32, p = 0.96 Unknown: HR 1.02, 95% CI 0.76–1.37, p = 0.88	
FVC	FVC < 50%: HR 1.29, 95% CI 1.06–1.57, p = 0.01* Unknown: HR 1.17, 95% CI 0.98–1.40, p = 0.09	FVC < 50%: HR 1.19, 95% CI 0.97–1.47, p = 0.09 Unknown: HR 0.92, 95% CI 0.40–2.11, p = 0.84
Mechanical ventilation	HR 5.05, 95% CI 2.39–10.69, p < 0.001*	HR 4.51, 95% CI 1.53–13.30, p = 0.006**
Double Lung Preference	HR 1.17, 95% CI 1.01–1.37, p = 0.04*	HR 1.34, 95% CI 1.09–1.66, p = 0.007**

Initial variables included in the univariable cox regression included pulmonary hemodynamics, comorbid conditions, and other variables that comprise the CAS score

*6MWD* 6-min walk distance. *BMI* Body mass index. *CAD* Coronary artery disease. *CAS* Composite lung allocation score. CI: Cardiac index. *CO* Cardiac output. *FEV1* Forced expiratory volume in 1 s. *FVC* Functional vital capacity. *mPAP* Mean pulmonary artery pressure. *O2* Oxygen. *PaCO2* Partial pressure of carbon dioxide. *PASP* Pulmonary artery systolic pressure. *PAWP* Pulmonary artery wedge pressure. *PVR* Pulmonary vascular resistance

*Variable with significant association with outcome of waitlist death in univariable cox regression (p < 0.05) and utilized in subsequent multivariable cox regression for waitlist death

**Variable with significant and independent association with waitlist death (p < 0.05) in multivariable cox regression

When accounting for time spent on waitlist (HR 1.04, 95% CI 1.03–1.05, p < 0.001), logistic regression still showed PVR > 5 WU (HR 1.68, 95% CI 1.12–2.53, p = 0.01) as well as PVR ≥ 3.9 WU (HR 1.45, 95% CI 1.08–1.95, p = 0.01) to be significantly and independently associated with waitlist mortality.

Multicollinearity analysis was performed to assess the degree of correlation between PVR, PASP, 6MWD, FVC, and oxygen requirement (Additional file 1: Table S1). All variables had a variance inflation factor between 1 and 3, suggesting a moderate correlation between them that likely does not impact the reliability of the multivariable regression findings.

## Discussion

The latest ESC/ERS guideline definitions for PH have prognostic implications with regards to patients with COPD-PH listed for lung transplantation. While patients with COPD and pre-capillary PH did not have decreased waitlist survival, those with COPD and severe PH, defined as PVR > 5 WU, did have decreased waitlist survival. Both severe PH, defined as PVR > 5 WU, and PVR ≥ 3.9 WU, are independently and significantly associated with increased risk of waitlist death, even when accounting for other pulmonary hemodynamic parameters and other factors known to influence transplant waitlist mortality.

Patients with COPD and severe PH were found to have worse transplant-free survival than those with COPD and moderate PH in an analysis of the Comparative, Prospective Registry of Newly Initiated Therapies for Pulmonary Hypertension (COMPERA) registry [9]. The guideline definitions of severe PH were updated in 2022, with a shift from an mPAP-focused definition to a PVR-focused definition, with PVR > 5 WU considered to be severe PH in chronic lung disease [7]. This determination was in part influenced by Zeder et al.’s study to determine prognostically relevant hemodynamic thresholds for severe PH in COPD [10]. After adjusting for age, sex, and FEV1, PVR > 5 WU was the best prognostic cut-off, demonstrating decreased 1-year survival as compared to mPAP. However, this study excluded patients undergoing lung transplantation [10]. To our knowledge, this is the first study using the new 2022 criteria for PH in chronic lung disease and lung transplantation to evaluate waitlist outcomes. While Nathan et al. examined the impact of PH defined as mPAP > 20 mmHg on patients listed for lung transplantation in the UNOS database, their analysis was prior to the new PVR cut-offs for pre-capillary and severe PH [11]. Waitlist survival was significantly worse among patients with COPD and severe PH as compared to patients with COPD and non-severe PH, suggesting that elevated PVR > 5 WU can effectively prognosticate lung transplant waitlist outcomes.

We additionally found that PVR ≥ 3.9 WU was independently and significantly associated with increased risk of transplant waitlist mortality among this cohort of patients with COPD-PH via cox regression after determining quintiles for the PVR distribution. While severe PH was associated with increased risk of waitlist mortality, our findings suggest that milder PH can also influence lung transplant waitlist outcomes in patients with COPD-PH. Given the significance in regression analysis, PVR may be a better prognostic pulmonary hemodynamic variable than mPAP in patients with COPD-PH. Further studies are required to fully elucidate the significance of milder levels of PH as determined by mPAP and/or PVR, particularly when taking into account the new diagnostic criteria for PH in chronic lung disease.

Notably, patients with COPD and pure pre-capillary PH, which now includes patients with PVR > 2 WU, did not have worse transplant waitlist survival. When considering the prior definition of pre-capillary PH, a subset of patients with COPD-PH have severe pre-capillary PH and poor survival despite having only moderate airflow obstruction [12]. However, such poor outcomes may not be as apparent when considering the new definition of pre-capillary PH in patients with COPD-PH. A recent, single-center study utilizing the 2022 ESC/ESR PH guideline definitions also found that among patients with COPD-PH, those with pure pre-capillary PH did not have worse transplant-free survival than patients with COPD-PH and other categories of PH [13]. The reason for this could be multifactorial as it has been shown that attempting to differentiate between PH associated with comorbid COPD and PH due to COPD with a pulmonary vascular phenotype is difficult, which could lead to heterogeneous outcomes [14]. Additionally, the change in PVR criteria to 2 WU for pre-capillary PH was in part based on a study of healthy patients, which found that the upper limit of normal PVR is less than 2 WU; this may not necessarily apply to patients with lung disease such as COPD [15]. While further studies examining the clinical and prognostic significance of the latest pre-capillary PH definition is necessary, caution should be used when considering this with respect to lung transplant waitlist prognosis in patients with COPD-PH.

In addition to PVR, age, BMI, PASP, 6MWD, CAD, hypertension, mechanical ventilation, blood type, and double lung preference also had significant and independent associations with transplant waitlist mortality. The significant association of age, BMI, PASP, 6MWD, and mechanical ventilation is not unexpected, as these are all patient characteristics accounted for in the 1-year waiting list mortality without a transplant aspect of the CAS [8]. BMI and 6MWD specifically are also components of the body mass index, degree of airflow obstruction, dyspnea, and exercise capacity (BODE) score, with lower BMI and 6MWD contributing to higher BODE score; each quartile increase in the BODE score yields an increased risk of mortality in patients with COPD [16]. We found that as BMI and 6MWD increased, the risk of transplant waitlist mortality decreased among patients with COPD-PH. Notably, we did not find FEV1 to be associated with transplant waitlist mortality. This is likely a result of missing spirometry data in the cohort database.

CAD, hypertension, blood type, and double lung preference were the non-CAS variables associated with transplant waitlist mortality in this cohort of COPD-PH patients. The presence of CAD and hypertension were each significantly associated with increased risk of waitlist mortality, which reflects the known association between cardiovascular disease and COPD. Patients with COPD have higher rates of CAD, and cardiovascular disease is a significant contributor to mortality in patients with COPD [17, 18]. This relationship is even evident amongst patients with severe enough COPD and PH to warrant lung transplantation evaluation and listing.

We found that blood type A was significantly associated with reduced risk of transplant waitlist mortality. A prior retrospective analysis of the OPTN registry found that candidates with blood type O experienced lower rates of lung transplantation and higher rates of waitlist mortality than matched blood type non-O patients [19]. Our findings highlight the continued significance of patient blood type. We also found that double lung preference was significantly associated with increased waitlist mortality among this cohort. This is consistent with prior studies; as patients with COPD restricted to double lung preference had significantly increased waitlist mortality [20]. Blood type and restricted double lung preference particularly may affect time spent on the waitlist, which in turn influences waitlist mortality; we found that those who died on the waitlist spent a significantly longer time on the waitlist. It is important to note there are additional factors that influence waitlist time and, in turn, mortality that we were unable to account for, such as pre-transplant immunization and compatible panel reactive antibody scores.

Even when accounting for comorbid conditions, CAS factors, and other factors known to influence transplant waitlist mortality, severe PH as defined by PVR > 5 WU and milder PH determined by PVR ≥ 3.9 WU are still significantly and independently associated with an increased risk of waitlist mortality. The only pulmonary hemodynamic variable incorporated into the CAS in patients listed for lung transplantation is PASP. However, we found that both PVR cut-offs were stronger predictors of transplant waitlist mortality than PASP among patients with COPD-PH. The CAS, formerly called the LAS, was implemented in 2005 to ensure the efficient and equitable allocation of lungs to active transplant candidates by considering and targeting pre-transplant waitlist mortality and post-transplant survival [21]. As such, the CAS incorporates a series of pre-transplant clinical data that has been shown to be predictive of pre- and post-transplant outcomes, such as PASP. [21] However, while the LAS/CAS system has been effective in improving pre- and post-transplant outcomes, it has been noted that it is not a sensitive predictor of waitlist mortality among patients with PH [22]. Given our findings detailing the significance of PH classified by PVR cut-off among patients with COPD-PH, incorporating PVR into the CAS may better identify patients with lung disease and PH at higher risk of waitlist death. Of course, additional studies examining the significance of severe PH and PVR among all lung diseases and pre- and post-transplant outcomes are necessary.

The principal strength of our study is that it utilizes a national all-inclusive registry of patients listed for lung transplantation. To our knowledge, this is the largest study evaluating patients with COPD-PH listed for lung transplantation and the significance of the latest ESC/ESR PH definitions in terms of waitlist outcomes, specifically the newest PVR cut-offs. Limitations included the study’s retrospective nature and limitations in the database; unfortunately, the database did not include candidate CAS values or a fully comprehensive collection of candidate testing data points, including important variables such as oxygen requirement and lung function. Most variables were collected at the time of listing and not at the time of death or transplant. The database also does not have data informing the parenchymal involvement of these patients, nor does it have data with regards to pulmonary vasodilator therapy in these patients. It is also important to note that these findings may not be applicable to those for whom lung transplant is contraindicated, such as those with poorly controlled dysfunction of another organ system or uncorrected CAD not amenable to revascularization, among other conditions [23]. However, the significance of the latest ESC/ESR definition of severe PH in lung disease with regards to transplant waitlist mortality among patients with COPD-PH is still notable and in line with mortality findings that led to the shift from mPAP to PVR when defining severe PH in chronic lung disease [10, 24].

The latest ESC/ERS guidelines for the diagnosis and treatment of PH established in 2022 shifted from mPAP to PVR when defining severe PH in patients with chronic lung disease, utilizing a PVR cut-off of 5 WU. We found that patients with COPD-PH listed for lung transplantation with a PVR > 5 WU had increased transplant waitlist mortality. Severe PH was significantly and independently associated with increased risk of waitlist mortality, even when accounting for comorbid conditions, patient variables incorporated into the CAS, and other factors that are known to influence transplant waitlist outcomes. Of the pulmonary hemodynamic variables, PVR had the strongest association with waitlist mortality among patients with COPD-PH. These findings suggest that incorporating PVR cut-offs may better identify patients with COPD-PH at increased risk of transplant waitlist mortality and can guide clinicians caring for these patients to refer them for lung transplantation evaluation.

### Supplementary Information

**Additional file 1: Table S1**. Multicollinearity analysis.

## Data Availability

The data that support the findings of this study are available on request from the SRTR. The data are not publicly available due to privacy or ethical restrictions.

## Abbreviations

6MWD: 6 Minute walk distance
BMI: Body mass index
BODE: Body mass index, degree of airflow obstruction, dyspnea, and exercise capacity
CAD: Coronary artery disease
CAS: Composite allocation score
CO: Cardiac output
COMPERA: Comparative, Prospective Registry of Newly Initiated Therapies for Pulmonary Hypertension
COPD: Chronic obstructive pulmonary disease
CO: Cardiac output
CT: Computed tomography
ESC: European Society of Cardiology
ERS: European Respiratory Society
FEV1: Forced expiratory volume in 1s
FVC: Functional vital capacity
GOLD: Global Initiative for Chronic Obstructive Lung Disease
HHRI: Hennepin Healthcare Research Institute
HRSA: Health Resources and Services Administration
mPAP: Mean pulmonary artery pressure
OPTN: Organ Procurement and Transplantation Network
PaCO2: Partial pressure of carbon dioxide
PASP: Pulmonary artery systolic pressure
PAWP: Pulmonary artery wedge pressure
PH: Pulmonary hypertension
PVR: Pulmonary vascular resistance
SRTR: Scientific Registry of Transplant Recipients
UNOS: United Network for Organ Sharing

## Declarations

### Guarantor statement

Shameek K. Gayen is the guarantor of the content of the manuscript, iand analysis.
