# Supplementary material for: Elevated pulmonary vascular resistance is associated with increased lung transplant waitlist mortality among patients with chronic obstructive pulmonary disease and pulmonary hypertension: a retrospective cohort analysis
Source: Respir Res. 2024 Feb 7;25:79. doi: 10.1186/s12931-024-02674-9 (PMC10848503; doi:10.1186/s12931-024-02674-9)
Supplement: Supplementary file 1 — Additional file 1: Table S1. Multicollinearity analysis. [file 12931_2024_2674_MOESM1_ESM.docx]

Additional file 1: Table S1: Multicollinearity Analysis

| Variable | Variance Inflation Factor |
| --- | --- |
| PASP | 1.39 |
| PVR | 1.37 |
| Oxygen requirement | 1.02 |
| 6MWD | 1.06 |
| FVC | 1.05 |

Given these variables were utilized in multivariable cox regression and may be interrelated, multicollinearity analysis was performed. All variables had a variance inflation factor greater than 1, suggesting moderate correlation, but less than 3, suggesting this correlation does not affect the significance of the multivariable cox regression.

6MWD: 6 minute walk distance
FVC: Functional vital capacity

PASP: Pulmonary artery systolic pressure

PVR: Pulmonary vascular resistance
